# Are There Sex-Specific Differences in Response to Adjunctive Host-Directed Therapies for Tuberculosis?

**DOI:** 10.3389/fimmu.2020.01465

**Published:** 2020-07-07

**Authors:** Noton K. Dutta, Bianca E. Schneider

**Affiliations:** ^1^Center for Tuberculosis Research, Department of Medicine, Johns Hopkins University School of Medicine, Baltimore, MD, United States; ^2^Junior Research Group Coinfection, Priority Research Area Infections, Research Center Borstel - Leibniz Lung Center, Borstel, Germany

**Keywords:** *Mycobacterium tuberculosis*, tuberculosis, host directed therapy, statins, treatment outcome, sex-specific differences, male-bias, mouse models

## Introduction

Tuberculosis (TB) appears to afflict men more than women, but the underlying reasons for this disparity and whether there are sex-based differences in TB treatment responses are unknown. Based on a knowledge gap in previously published studies, a pertinent research question is being asked: Are there sex-specific differences in response to adjunctive host-directed therapy (HDT) for TB? Using statins as a prototype, we also highlight the incorporation of appropriately designed studies on sex differences in HDT.

There is an important need for new drugs to help control the TB pandemic, counteract the emergence of drug resistance worldwide, and supplement the limited number of antimicrobials that are currently in the TB drug discovery pipeline. Therefore, the repurposing of existing drugs, including host-directed therapies (1), is being investigated as a means of accelerating the development of novel TB regimens in the clinical setting. We hypothesize that there is a sex difference in responses to adjunctive HDT because males are more susceptible to TB and various HDTs may have differential, sex-dependent impacts on inflammation. This question is important in the context of ongoing clinical trials that are investigating the potential roles of various HDTs as adjunctive therapy for TB, including the lipid-lowering agents, statins (StAT-TB, NCT03882177), the hypoglycemic agent, metformin (CTRI/2018/01/011176), and the anticancer agent, imatinib (IMPACT-TB, NCT03891901). Mayer-Barber and colleagues reported that the 5-lipoxygenase inhibitor zileuton, which is FDA-approved for the treatment of asthma, increased prostaglandin E2 (PGE2) levels in the lungs of *Mycobacterium tuberculosis*-infected mice and prevent acute mortality, while decreasing pulmonary bacillary loads, tissue necrosis, and type I IFN levels (2). Data from Pace et al. suggested that zileuton is less effective in males, prompting consideration of sex-based differences in leukotriene biosynthesis blockade for respiratory and cardiovascular diseases (3).

## Increased TB Incidence in Men

Despite over a century of research, TB still kills ~1.5 million people every year (4). Globally, TB notification data show a male-to-female ratio of bacteriologically-confirmed pulmonary TB ranging from 1.2 in Ethiopia to 4.9 in Viet Nam (4), but the underlying reasons for this male bias remain elusive. Presumably, both gender- and sex-related factors contribute to higher TB rates in men. Socioeconomic and cultural factors influence exposure and help-seeking behavior, and confounding factors, such as smoking, alcohol and drug use, which are known risk factors for TB, are more common in men (5, 6). However, these factors are unlikely to explain the consistent global male bias, and biological differences between the sexes likely affect susceptibility to mycobacterial infection and disease outcome (7, 8). Despite the well-known sex-based differences in human TB incidence rates, due to practical considerations, most animal studies have either used only one sex or do not report the sex of the animals at all.

## The Role of Biological Sex in TB

Recent data suggest that male C57BL/6 mice are less able to control *M. tuberculosis* infection relative to their female counterparts due to impaired innate and adaptive immune responses, as manifested by increased lung bacillary burdens and accelerated death (9). Divergent innate immune priming events occurring early in an infection influence both the bacterial load “set-point” and the ensuing adaptive immune response (10).

Innate recognition of pathogens and the induction of inflammatory and antimicrobial immune responses differ between the sexes (11). Factors that have been shown to account for the sex-based disparity in immune responses include genetic factors and hormonal mediators. Estrogen was shown to boost the ability of macrophages to kill *Streptococcus pneumoniae* (12), and the antimicrobial activity of peritoneal macrophages from female mice was significantly more potent against *Mycobacterium intracellulare* than that of male mice (13). IFNγ, a critical cytokine mainly produced by CD4^+^ and CD8^+^ T cells, increases the anti-bacterial effector functions of macrophages (14). Invariant natural killer T (iNKT) cells are a subset of T cells that bridge innate and adaptive immunity, recognize *M. tuberculosis*-infected macrophages, produce IFNγ, and kill intracellular bacteria (15). Estradiol induces iNKT cells from female mice to produce more IFNγ than their male counterparts (16). Moreover, IFNγ production by iNKT cells is impaired by female castration or genetic ablation of the estradiol receptor, and the normal phenotype is restored by estradiol injection (16, 17) In contrast, testosterone was shown to exert anti-inflammatory effects in macrophages and other innate immune cells that express the androgen receptor (AR), by inhibiting pro-inflammatory factors such as TNF-α and nitric oxide (NO), and increasing the synthesis of interleukin (IL)-10 (18). Male castration also increases TNF-α secretion in macrophages (19). These results illustrate the general, but probably simplistic, perception of estradiol as an immunity-sustaining or immunity-enhancing mediator, and of testosterone as a mediator that inhibits the immune response. Similarly, male mice are more susceptible to TB than female mice, which can be prevented by male castration (20), suggesting that testosterone could be a TB susceptibility factor. Sex hormones not only regulate innate but also adaptive responses. Generally, females show higher T cell activity including the production of T_h_1-type cytokines such as IFNγ, and greater antibody responses than males (11). Differences in adaptive immunity might contribute to the impaired long-term containment of *M. tuberculosis* observed in mice (9, 20, 21). Indeed, we recently showed that increased male susceptibility in *M. tuberculosis* infection is associated with reduced B cell follicle formation in the lung (21).

Sex differences in TB can be mediated by more than hormonal influence. The X chromosome expresses a number of immune-related genes, as well as a number of immune-associated microRNAs (11). Females have two X chromosomes and benefit from a genetic diversity due to cellular mosaicism and genes escaping X chromosome inactivation which is often advantageous because it ameliorates the deleterious effects of X-linked mutations. Thus, sex disparity in TB may be a result of both hormonal and genetic influences.

## Statins as Adjunctive HDT for TB

In addition to their cholesterol-lowering properties, β-Hydroxy β-methylglutaryl-CoA (HMG-CoA) reductase inhibitors (statins) have been shown to have broad anti-inflammatory and immunomodulatory properties (22, 23), and their use has been associated with significantly decreased risk of TB (24). Statins have been shown to have antimicrobial and immunomodulatory activity in mouse models of infection against intracellular pathogens, including *Salmonella enterica* and *Chlamydia pneumoniae*. Statins' mode of action in antimicrobial therapy is centered on controlling the infection rate in macrophages and monocytes. Mature macrophages respond to diverse environmental signals by expressing many functional phenotypes, from the *classical* phenotype (M1, proinflammatory) to the *alternative* phenotype (M2, anti-parasitic, immunoregulatory). Early secreted antigenic target of 6-kDa of *M. tuberculosis* induces M1 phenotype in the early stage and then polarizes to M2 phenotype in the later stage of TB infection (25). Heat-Shock Protein 16.3 of *M. tuberculosis* induces M2 polarization in the mouse bone marrow-derived macrophage model via CCRL2/CX3CR1 and may be mediated by the AKT/ ERK/p38-MAPK signaling pathway. In fact, adoptive transfer of M2 macrophages is effective in controlling TB infection apart from its role in controlling tissue damage (26)

In a recent publication (27), we screened eight different statins for a cytotoxic effect, anti-tubercular activity, synergy with first-line drugs in macrophages, pharmacokinetics, and adjunctive bactericidal activity in two different mouse models as a potential adjunctive therapy to existing first-line TB drugs (27). Pravastatin exhibited the most favorable therapeutic index *in vitro* and better anti-TB activity in the standard BALB/c mouse model and in the C3HeB/FeJ mouse model of human-like necrotic TB lung granulomas (27). Previous studies already demonstrated that pravastatin modulated phagosomal maturation characteristics in macrophages via phenocopying macrophage activation, and its use as an adjunctive agent in chronically infected mice altered lung and peripheral immune responses (27, 28). We also found that simvastatin adjunctive therapy enhanced the first-line TB regimen's antimicrobial activity and shortened the time required to achieve cure in a BALB/c mouse model of chronic TB infection (29). Bruiners et al. recently demonstrated that simvastatin inhibits mechanistic target of rapamycin complex 1 (mTORC1) activity and regulates transcription factor EB (TFEB) nuclear translocation to induce autophagy and lysosomal biogenesis (30). In addition, statins show synergy with the key sterilizing drug, rifampin. Retrospective cohort studies found that statin use was associated with a reduced incidence of active TB disease (31, 32). However, studies focusing on anti-TB effect of statin on the basis of sex of the patients are lacking. Also, the preclinical studies were conducted exclusively in female mice, and it is unknown whether the adjunctive, host-directed anti-TB properties of statins are sex-specific, which is an important consideration for their potential clinical utility.

## Sex Differences in the Effectiveness of Statins

Previous studies (33) have shown sex-based differences for statins with respect to mortality following myocardial infarction (34) and in reducing the risk of Alzheimer's disease (35). These effects may result from differences in drug metabolism, but hormonal effects have not been explored. In some animal studies, the simvastatin metabolism rate was found to be considerably higher in males than in females (36); this statin might, therefore, be expected to have a greater clinical effect in males. This hypothesis was not confirmed in studies that enrolled human volunteers, while, in contrast, a lower rate of simvastatin and lovastatin metabolism was observed in men than in women (37). Moreover, several epidemiological studies have reported greater statin-induced reductions in both LDL and total cholesterol in women than in men (38).

## Conclusions and Future Directions

**Figure 1:**
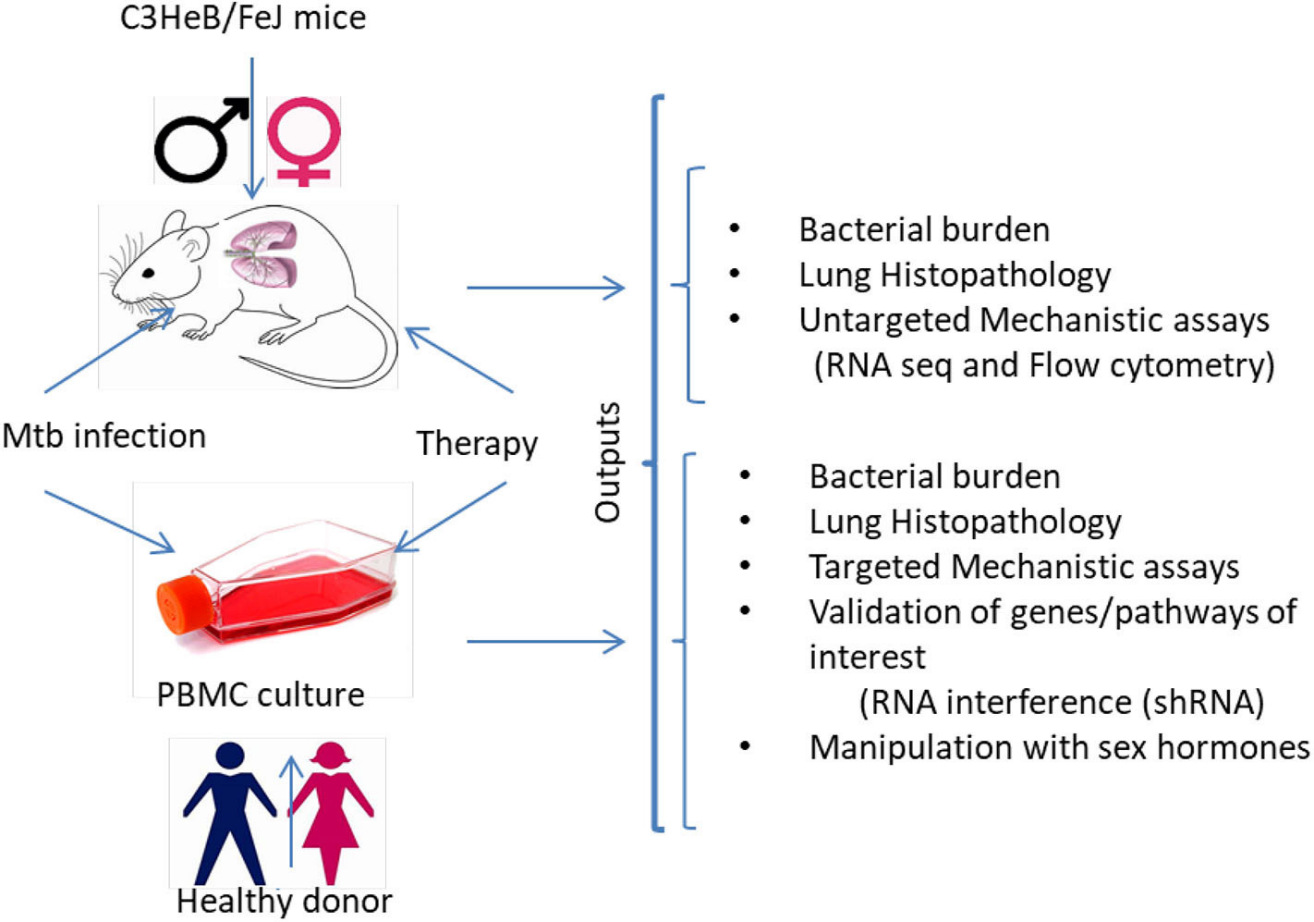
Experimental sketch for preclinical examination of sex-differences in responsiveness to HDTs. The experimental strategy would help to determine sex-based differences in clinical, microbiological, immunological and histopathological outcomes in *M. tuberculosis* infected (i) C3HeB/FeJ male and female mice and (ii) human monocyte-derived macrophages from healthy male and female donors before and after treatment with HDTs. Several untargeted and targeted approaches can be used to investigate the molecular mechanisms associated with the sex-specific anti-TB activity of HDTs. MDMs, monocyte-derived macrophages; shRNA, Small hairpin RNA.

It has already been established that sex must be considered in preclinical studies, although clearly defined “go/no-go” endpoints to justify further testing of various HDT agents in clinical trials have yet to be defined. It is unknown if the anti-TB activity of statins and other promising HDTs are sex specific. The study of animals and cells of both sexes is essential to include preclinical study designs that will control drug exposure, efficacy, metabolism, and immune response variabilities on HDT for TB. Here, we propose to understand if sex influences the adjunctive anti-TB activity and immune responses of HDTs in: (i) a murine model of chronic TB infection with human-like necrotic lung granulomas; and (ii) *ex vivo* infection of human monocyte-derived macrophages (MDMs) (Figure 1). *M. tuberculosis* residing in necrotic mouse lung lesions may be more akin to persisters in human lesions with a reduced response to direct-acting anti-TB drugs; further, these areas represent relative pharmacological sanctuaries. Because of these favorable features, we and other groups have begun to use C3HeB/FeJ mice to test the efficacy of various antitubercular regimens and novel anti-inflammatory therapies (27, 39). An MDM system was chosen because macrophages are the key cell type harboring *M. tuberculosis* during infection and because this model is amenable to testing under multiple, controlled perturbations. Moreover, circulating monocytes are natural precursors of lung tissue-resident macrophages. In addition, using primary cells, researchers will be able to validate the key findings *in vivo* in macrophages from both males and females by applying RNA interference technology. Downstream “omics” data will provide the opportunity to investigate the mechanisms underlying sex-based differences in host control of *M. tuberculosis* infection, as well as potential differences in response to standard antitubercular therapy and HDT between the sexes. Such results will improve the predictive value of animal models to evaluate treatment efficacy by HDT agents with respect to variables such as sex and potential clinical utility of particular immunomodulators. The identification of the biological pathways underlying sex differences in HDTs for TB will play an essential role in the development of more effective personalized healthcare. One unanswered question is to what extent the mouse model will be predictive of HDTs for TB in humans.

## Author Contributions

ND and BS have made substantial, direct and intellectual contribution to the work, critically reading an earlier version of this manuscript, and approved it for publication. All authors contributed to the article and approved the submitted version.

## Conflict of Interest

The authors declare that the research was conducted in the absence of any commercial or financial relationships that could be construed as a potential conflict of interest.
